# Non-invasive Gamma Brain Wave Optimization (BWO-G) for Cognitive and Emotional Recovery in an Adolescent: A Case Study on Radio Electric Asymmetric Conveyer (REAC) Neuro Psycho Physical Optimization (NPPO) BWO-G Treatment

**DOI:** 10.7759/cureus.72819

**Published:** 2024-11-01

**Authors:** Valeria Modesto', Arianna Rinaldi, Vania Fontani, Salvatore Rinaldi

**Affiliations:** 1 Department of Research, Rinaldi Fontani Foundation, Florence, ITA; 2 Department of Regenerative Medicine, Rinaldi Fontani Institute, Florence, ITA; 3 Department of Adaptive Neuro Psycho Physio Pathology and Neuro Psycho Physical Optimization, Rinaldi Fontani Institute, Florence, ITA

**Keywords:** adolescent mental health, cognitive dysfunction, cognitive enhancement, gamma brain waves, gender dysphoria, mental health rehabilitation, neuromodulation, quantitative eeg, reac

## Abstract

This case report describes the application of the radio electric asymmetric conveyer (REAC) gamma brain wave optimization (BWO-G) treatment in a 16-year-old female patient with a history of emotional trauma, depressive symptoms, and gender dysphoria. The patient underwent 18 sessions of REAC Neuro Psycho Physical Optimization (NPPO) BWO-G, with quantitative electroencephalography (QEEG) conducted pre- and post-treatment. Initial QEEG analyses indicated altered brain wave activity, with peaks in alpha rhythm frequencies in occipital and right posterior temporal areas. Following treatment, significant improvements were observed, including a reduction in delta and theta rhythms and increased alpha and gamma rhythms, corresponding to enhanced cognitive clarity and emotional regulation. Clinically, the patient experienced reduced anxiety, improved mood, heightened social engagement, better auditory tolerance, and resumed weight management. These findings suggest that the REAC NPPO BWO-G treatment may be an effective, non-invasive approach to enhancing cognitive and neuropsychological resilience in individuals with complex psychosocial challenges.

## Introduction

Gamma brain waves, typically characterized by frequencies ranging from approximately 30 to 100 Hz, are closely associated with high-level cognitive functions, including attention, memory, and consciousness [1]. Alterations in gamma rhythms can profoundly impact brain function and overall mental health [2]. Elevated gamma activity has been linked to enhanced cognitive processes [3] such as improved focus, faster information processing, and better working memory [4], which are beneficial across various contexts, from academic performance to rapid decision-making in high-stress environments.

On the other hand, disruptions in gamma wave activity have been implicated in several neurological and psychiatric disorders. For instance, abnormal gamma rhythms have been observed in individuals with schizophrenia, Alzheimer's disease [2], and autism spectrum disorder [5], highlighting the critical role these waves play in maintaining cognitive coherence and neural synchrony. In schizophrenia, reduced gamma activity is believed to contribute to the cognitive deficits and sensory processing abnormalities that characterize the disorder [6].

Therapeutic interventions targeting gamma brain wave activity, such as neurofeedback, transcranial alternating current stimulation (tACS) [7], transcranial magnetic stimulation (TMS) [8], and specific pharmacological agents, show potential for treating conditions associated with gamma wave dysregulation. More recently, emerging treatments like the radio electric asymmetric conveyer (REAC) gamma brain wave optimization (BWO-G) have been developed to enhance gamma activity.

The REAC Neuro Psycho Physical Optimization (NPPO) [9] BWO-G protocol was designed to optimize gamma brain wave generation, which is associated with heightened cognitive activity and motor strategies. This neuromodulation protocol aims to restore neuronal allostasis, thereby improving overall functionality.

The treatment is administered using an asymmetric conveyer probe (ACP) applied to the cervico-brachial area, connected to the REAC device (BENE Mod. 110, ASMED SRL, Scandicci, Italy). This procedure is entirely non-invasive, with no subjective sensation experienced by the patient. Each session lasts approximately five minutes, totaling 18 sessions over six days, with three sessions per day. An interval of at least one hour was maintained between sessions to ensure adequate treatment spacing.

This approach could offer novel methods for enhancing cognitive performance in healthy individuals and rehabilitating cognitive functions in those with neurological impairments. Understanding and modulating gamma brain rhythms thus represents a promising avenue for advancing clinical practice and cognitive enhancement strategies.

In this manuscript, we present the results from a case report of a patient who underwent REAC NPPO BWO-G treatment, with quantitative electroencephalography (QEEG) monitoring performed before and after treatment.

## Case presentation

Patient information

The patient is a 16-year-old female with a history of premature birth at 34 weeks gestation. During her early childhood, she exhibited irritability and had difficulty adjusting to the school environment. At the age of four, she underwent surgical interventions to address recurrent middle ear infections. Her emotional history includes significant trauma at age 11, when she experienced the death of a pet, the passing of her grandmother, and her parents' divorce. These events triggered the onset of depressive symptoms, gender dysphoria, and social withdrawal.

Clinical findings

The patient displayed several psychological and emotional challenges, including depressive symptoms, social isolation, and significant gender dysphoria. She also exhibited auditory hypersensitivity, particularly to specific "wet noises," which exacerbated her discomfort in social situations. Over the course of four years, the patient experienced a 30 kg weight gain and showed a strong aversion to recalling her childhood experiences. Previous psychological counseling from ages four to 10 and again at age 14 failed to alleviate her symptoms.

Timeline

The patient's history includes premature birth, medical interventions for middle ear infections at age four, emotional trauma at age 11, and unsuccessful psychological counseling from ages four to 10 and again at age 14. By age 16, she was referred for REAC NPPO BWO-G treatment for cognitive and emotional recovery.

Therapeutic assessment and intervention

Before starting the REAC NPPO BWO-G treatment, a comprehensive QEEG analysis was performed [10], which revealed altered brain wave activity with pronounced peaks in alpha rhythm frequencies in the occipital and right posterior temporal regions. The REAC NPPO BWO-G protocol was then administered using an ACP applied to the cervico-brachial area. Each session lasted approximately five minutes, with a total of 18 sessions conducted over six days, at a rate of three sessions per day. Each session was spaced at least one hour apart to ensure adequate intervals between treatments.

Post-treatment QEEG analyses indicated a marked reduction in delta and theta rhythms in the temporal areas, accompanied by a decrease in the alpha rhythm in the occipital region. These findings corresponded with significant clinical improvements in anxiety, mood, cognitive clarity, and social engagement.

EEG Power Spectra Analysis

**Figure 1:**
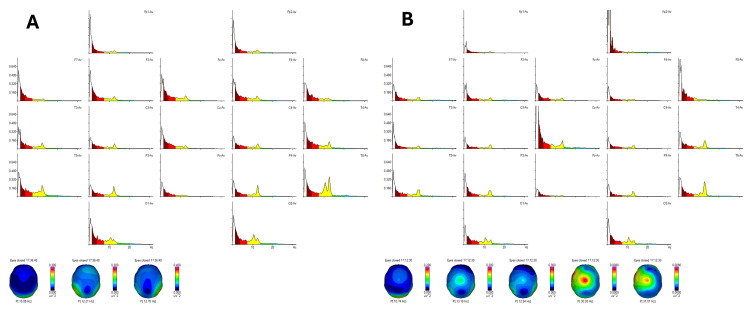
EEG power spectra analysis (fragment: eyes closed 17:36:48, offset: 0.00 s, length: 299.98 s, number of epochs: 144) Figure 1A demonstrates the pre-treatment peak in alpha rhythm frequency at 10.99 Hz in the occipital region and 12.70 Hz in the right posterior temporal areas, with diffuse delta and theta rhythm distribution. Figure 1B shows the post-treatment decrease in alpha rhythm to 10.74 Hz, correlating with reduced anxiety and enhanced mental clarity. The emergence of gamma rhythm activity in the central region is indicative of improved cognitive function. EEG: electroencephalography

The initial EEG power spectra analysis [11] revealed a peak in alpha rhythm frequency at 10.99 Hz in the occipital region and 12.70 Hz in the right posterior temporal areas, with delta and theta rhythms being diffusely distributed (Figure 1A). Post-treatment EEG analysis demonstrated a significant change, with the posterior alpha rhythm frequency decreasing to 10.74 Hz (Figure 1B). This reduction correlated clinically with improvements in anxiety and cognitive clarity observed in the patient. Additionally, gamma rhythm activity emerged in the central region, extending bilaterally across both hemispheres, suggesting enhanced sensory-motor integration and cognitive coherence.

Comparative analysis of EEG power spectra for band ranges

**Figure 2:**
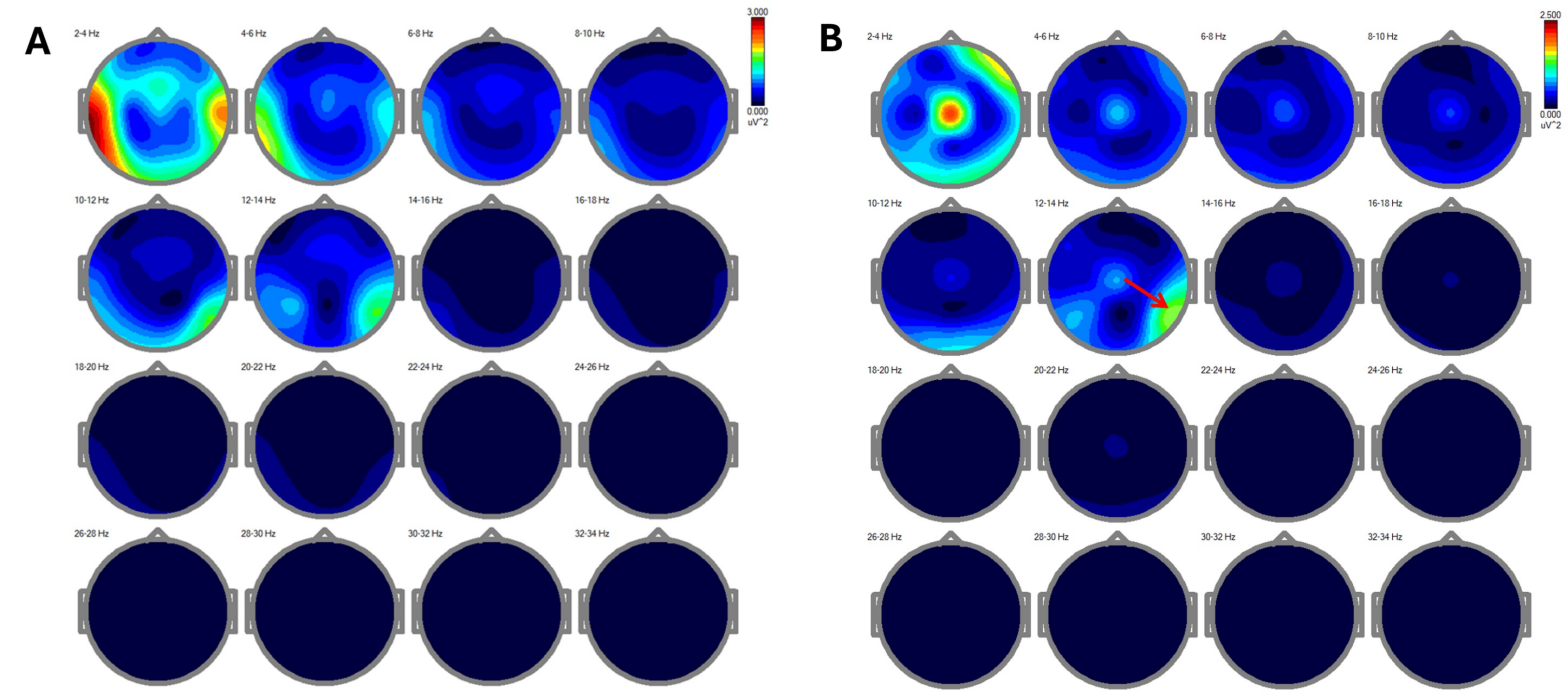
Frequency maps of EEG power spectra across different bands Figure 2A: Fragment: eyes closed at 17:36:48, offset: 0.00 s, length: 299.98 s, number of epochs: 144. Notable presence of delta rhythm in the temporal and mid-frontal regions. Figure 2B: Significant reduction of slow rhythms in the temporal areas. The red arrow highlights the rapid alpha rhythm. Note: Frequencies in the 2-4 Hz range are artifacts from eye movement and the Cz channel. EEG: electroencephalography

The comparative analysis of EEG power spectra across different frequency bands (delta, theta, alpha, beta, and gamma) [12] revealed substantial changes post-treatment. Initially, a notable presence of delta rhythms was observed in the temporal and mid-frontal regions (Figure 2A). Following REAC NPPO BWO-G treatment, there was a marked reduction of slow rhythms in these areas, with an evident shift toward faster alpha and gamma rhythms, particularly in the central and parietal regions (Figure 2B). This transition is consistent with improved cognitive processing and mental clarity.

Independent Component Analysis (ICA) with winEEG

**Figure 3:**
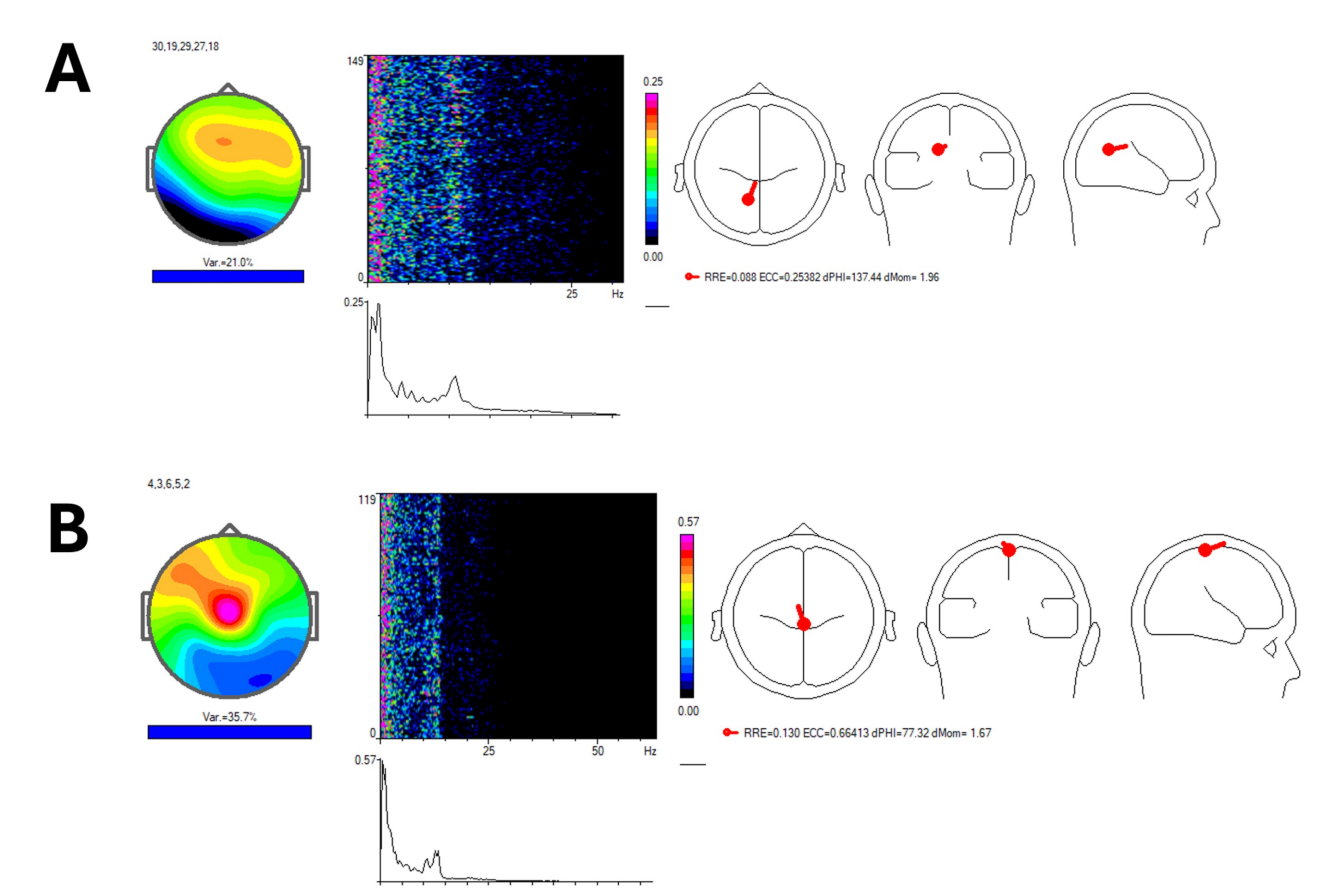
Independent component analysis with winEEG Figure 3A displays pre-treatment frequency peaks between 1.95 Hz and 10.74-12.45 Hz, highlighting a restricted range of brain wave activity. Figure 3B shows post-treatment frequency peaks extending up to 13.43 Hz, indicating a more dynamic and integrated brain wave activity pattern.

The ICA performed using winEEG [13] showed frequency peaks pre-treatment ranging from 1.95 Hz to 10.74-12.45 Hz (Figure 3A). Post-treatment, frequency peaks shifted to a range of 1.71-13.43 Hz, indicating a broader and more balanced distribution of brain wave frequencies (Figure 3B). This change signifies improved cognitive flexibility and processing speed, which aligns with the patient's reported improvements in mood, anxiety, and cognitive clarity.

Standardized Low-Resolution Brain Electromagnetic Tomography (sLORETA) Analysis

sLORETA analysis is a neuroimaging technique used to estimate the three-dimensional distribution of brain wave activity within different regions of the brain [14]. By providing a spatial representation of electrical activity, sLORETA allows researchers and clinicians to identify specific brain areas involved in various cognitive, sensory, and emotional processes. This technique offers valuable insights into how different brain regions are activated under certain conditions or in response to treatment.

**Figure 4:**
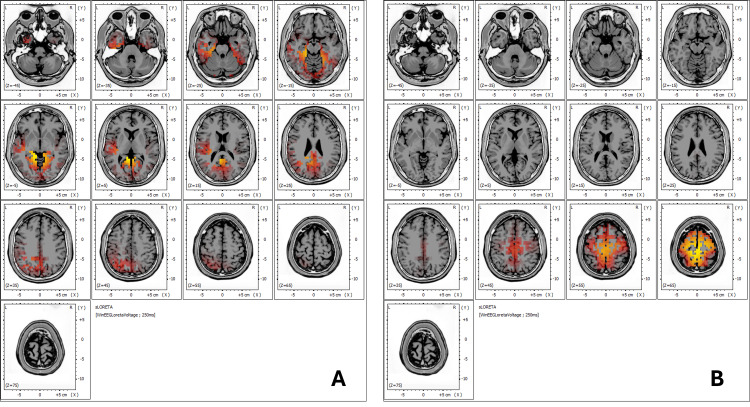
LORETA analysis Figure 4A: The patient exhibited brain wave activity across a spectrum of frequencies, ranging from slower waves (delta and theta) to faster frequencies, including an alpha rhythm at 12.45 Hz. These were observed in Brodmann areas 30, 19, 29, 27, and 18. These regions are mainly associated with functions such as emotional regulation, memory encoding (verbal semantic, face, image, auditory), working memory, facial emotional perception, emotional experience and regulation, and visual information processing. Figure 4B: Following treatment, a more widespread frequency distribution was noted in the motor and somatosensory cortex areas, with improvement in working memory processing, visuospatial attention, and motor execution. LORETA: low-resolution brain electromagnetic tomography

In Figure 4, the sLORETA analysis demonstrates notable changes in brain wave activity before and after treatment. Figure 4A shows that, prior to treatment, the patient exhibited brain wave activity across a range of frequencies, from slower waves (delta and theta) to faster frequencies, including an alpha rhythm at 12.45 Hz. These were localized in Brodmann areas 30, 19, 29, 27, and 18, which are linked to functions such as emotional regulation, receptive language, auditory processing, semantic analysis, visual information processing, and sentence formation.

After the treatment, as shown in Figure 4B, there was a more extensive distribution of frequencies across the prefrontal cortex, motor regions, and both the anterior and posterior cingulate gyrus. Notably, the slow delta and theta rhythms previously present in the temporal areas (Brodmann areas 21 and 42) were no longer observed. Clinically, this change correlated with improved tolerance to wet noises, which allowed the patient to integrate more comfortably into public environments, such as restaurants, indicating enhanced auditory processing and emotional regulation.

## Discussion

This case report demonstrates the potential of REAC NPPO BWO-G in achieving significant cognitive and emotional improvements in an adolescent with complex neuropsychological challenges, such as emotional trauma and gender dysphoria. The therapeutic effects were substantiated by changes in brain wave patterns, as revealed by QEEG analysis. These findings suggest that modulating gamma brain wave activity is key to enhancing cognitive coherence, emotional regulation, and sensory processing. This non-invasive treatment, which achieves brain modulation without discomfort, presents a promising alternative to existing neuromodulation methods.

Comparison with other neuromodulation techniques

Several neuromodulation techniques, including neurofeedback, tACS, and TMS, have been explored for their ability to influence brain wave activity and improve cognitive and emotional functioning. These techniques have shown efficacy in enhancing gamma wave activity and alleviating symptoms in conditions such as schizophrenia, autism, and Alzheimer's disease. However, the mechanisms through which they affect brain wave patterns and their effects on QEEG vary significantly compared to REAC NPPO BWO-G.

Neurofeedback allows individuals to self-regulate their brain wave activity by receiving real-time feedback on brain wave frequencies, typically aiming to increase gamma activity over time. While effective, it often requires prolonged training sessions and active patient participation, which may not be feasible for everyone. Furthermore, QEEG changes with neurofeedback tend to manifest more gradually [15], in contrast to the rapid improvements observed with REAC NPPO BWO-G, as evidenced in this case study.

tACS and TMS stimulate specific brain regions to influence gamma rhythms [16]. These techniques have been investigated for their role in treating neuropsychiatric disorders, where they are found to effectively increase gamma oscillations. However, these methods may involve discomfort, such as scalp irritation or mild side effects, which can reduce their appeal for certain patients. Moreover, QEEG changes resulting from tACS and TMS tend to be localized to the specific areas targeted by the stimulation, limiting their broader impact on brain wave activity [17].

In contrast, REAC NPPO BWO-G induces a more extensive modulation of brain wave activity without requiring patient engagement or invasive procedures. This technique restores neuronal allostasis, resulting in marked QEEG changes, such as the reduction in delta and theta rhythms and an increase in alpha and gamma rhythms, within a relatively short time frame of 18 sessions over six days. These QEEG improvements correlated with the patient's clinical progress, including enhanced cognitive clarity, emotional regulation, and sensory tolerance. The speed of these changes sets REAC NPPO BWO-G apart from other methods, offering a more efficient treatment option.

Clinical relevance of QEEG findings

The pre-treatment QEEG in this case revealed pronounced peaks in alpha rhythms, alongside diffuse delta and theta rhythms, in the occipital and right posterior temporal regions. Such patterns are commonly associated with dysregulated brain wave activity in individuals with neuropsychological disorders​ [18]. After REAC NPPO BWO-G treatment, a substantial shift was observed in the QEEG analysis, with a reduction in slower delta and theta rhythms and an increase in faster alpha and gamma waves, particularly in the central and parietal regions. These changes reflect enhanced cognitive processing, emotional stability, and improved sensory integration [19].

Compared to other neuromodulation techniques, the effects of REAC NPPO BWO-G on QEEG appear more widespread. For instance, TMS typically focuses on stimulating specific brain areas associated with cognitive and emotional functions [20], and while it can yield positive outcomes, the QEEG effects are often confined to those specific regions. In contrast, REAC NPPO BWO-G demonstrates broader changes in brain wave activity, suggesting it influences multiple interconnected brain networks simultaneously, which may lead to more comprehensive therapeutic effects.

Addressing gaps in knowledge

While this case study provides promising evidence for the efficacy of REAC NPPO BWO-G, further research is needed to confirm its long-term effects and broader applicability. Larger-scale, controlled studies could help clarify how this treatment compares to other neuromodulation techniques across a wider range of neuropsychological conditions. Moreover, the present study demonstrates significant changes in brain wave activity and clinical outcomes. This case report, along with other studies, has shown that these changes result from the optimization of endogenous bioelectrical activity, which is specifically targeted by the various therapeutic protocols of REAC technology.

Advancing the field

This case study highlights the value of REAC NPPO BWO-G as a non-invasive, effective, and rapid method for modulating brain wave activity, supported by significant changes in QEEG within a short duration. In comparison to other neuromodulation techniques, REAC NPPO BWO-G offers several advantages, including ease of use, accessibility, and broader modulation of brain activity. The findings suggest that REAC NPPO BWO-G could be a promising tool for enhancing cognitive and emotional resilience, particularly in patients with complex neuropsychological challenges.

## Conclusions

The integration of EEG power spectra, comparative analysis, ICA, and sLORETA findings in this case demonstrates the potential of REAC NPPO BWO-G to induce significant changes in brain wave dynamics, particularly in enhancing gamma rhythms and reducing slower delta and theta rhythms. These neurophysiological changes were accompanied by marked clinical improvements in cognitive clarity, emotional regulation, and sensory processing, supporting the hypothesis that non-invasive modulation of gamma activity can lead to broader neuropsychological resilience.

Compared to other neuromodulation techniques, REAC NPPO BWO-G offers distinct advantages in terms of non-invasiveness, ease of application, and comprehensive modulation of brain activity, as observed through QEEG changes. While these findings are promising, further research is needed to explore the long-term effects and broader applicability of this approach across different neuropsychological conditions and populations. This case suggests that REAC NPPO BWO-G could be an effective treatment for individuals with complex psychosocial challenges, but larger-scale studies are necessary to validate its therapeutic potential.
